# Improving protein order-disorder classification using charge-hydropathy plots

**DOI:** 10.1186/1471-2105-15-S17-S4

**Published:** 2014-12-16

**Authors:** Fei Huang, Christopher J Oldfield, Bin Xue, Wei-Lun Hsu, Jingwei Meng, Xiaowen Liu, Li Shen, Pedro Romero, Vladimir N Uversky, A Keith Dunker

**Affiliations:** 1 Center for Computational Biology and Bioinformatics, Department of Biochemistry and Molecular Biology, Indiana University School of Medicine, Indianapolis, Indiana, USA; 2 Department of Cell Biology, Microbiology, and Molecular Biology, University of South Florida. Tampa, Florida, USA; 3 Chemical and Biological Engineering, University of Wisconsin-Madison, Madison, Wisconsin, USA; 4 Department of Molecular Medicine, University of South Florida, Tampa, Florida, USA; 5 USF Health Byrd Alzheimer's Research Institute, Morsani College of Medicine, University of South Florida, Tampa, Florida, USA; 6 Institute for Biological Instrumentation, Russian Academy of Sciences, 142290 Pushchino, Moscow Region, Russia

**Keywords:** Intrinsically disordered proteins, natively unstructured or unfolded proteins, structure and disorder prediction, support vector machines

## Abstract

**Background:**

The earliest whole protein order/disorder predictor (Uversky et al., Proteins, 41: 415-427 (2000)), herein called the charge-hydropathy (C-H) plot, was originally developed using the Kyte-Doolittle (1982) hydropathy scale (Kyte & Doolittle., J. Mol. Biol, 157: 105-132(1982)). Here the goal is to determine whether the performance of the C-H plot in separating structured and disordered proteins can be improved by using an alternative hydropathy scale.

**Results:**

Using the performance of the CH-plot as the metric, we compared 19 alternative hydropathy scales, with the finding that the Guy (1985) hydropathy scale (Guy, Biophys. J, 47:61-70(1985)) was the best of the tested hydropathy scales for separating large collections structured proteins and intrinsically disordered proteins (IDPs) on the C-H plot. Next, we developed a new scale, named IDP-Hydropathy, which further improves the discrimination between structured proteins and IDPs. Applying the C-H plot to a dataset containing 109 IDPs and 563 non-homologous fully structured proteins, the Kyte-Doolittle (1982) hydropathy scale, the Guy (1985) hydropathy scale, and the IDP-Hydropathy scale gave balanced two-state classification accuracies of 79%, 84%, and 90%, respectively, indicating a very substantial overall improvement is obtained by using different hydropathy scales. A correlation study shows that IDP-Hydropathy is strongly correlated with other hydropathy scales, thus suggesting that IDP-Hydropathy probably has only minor contributions from amino acid properties other than hydropathy.

**Conclusion:**

We suggest that IDP-Hydropathy would likely be the best scale to use for any type of algorithm developed to predict protein disorder.

## Background

Intrinsically disordered proteins (IDPs) exist as flexible ensembles under normal physiological conditions, thus lacking stable tertiary structures, and yet carrying out various biological functions [1-4]. These IDPs challenge the universality of the sequence → structure → function paradigm, with biological functions associated instead with flexible ensembles rather than with structured proteins. IDPs are involved in numerous biological activities, such as providing sites for post-translational modifications, entropic spring-based restoring forces, flexible linkers, specific binding to multiple partners, multiple binding to a specific partner, and many others [5-15].

Many computational tools have been developed for predicting IDPs and IDP regions from amino acid sequence, including several Predictors of Natural Disordered Regions (PONDR^®^s) [16-19], IUPred [20,21], DisoPred [7,22], SPINE-D[23], FoldIndex[24] and more than 50 others [25,26]. For the various sequence-based approaches using machine learning methodologies, hydrophobicity is widely if not universally used as one of the inputs [16,20-24,26-29].

One of the more widely used prediction methods is based on a very simple model: repulsion from like charges favors unfolding while increased hydrophobicity favors folding [30]. In this approach, normalized net charge is plotted against normalized hydropathy, which is calculated from the hydropathy scale developed by Kyte-Doolittle (1982) [31], giving the charge-hydropathy (C-H) plot. Remarkably, this simple C-H plot largely separates IDPs from structured proteins [30]. This model has been used both for whole protein disorder prediction via the C-H plot [30] and for residue-by-residue disorder prediction via the FoldIndex algorithm [31].

The values for the original hydrophobicity scale were estimated experimentally as the side chain free energies of transfer from selected organic solvents to water [32]. The selected organic solvents, dioxane and aqueous ethanol, were chosen because their dielectric constants are similar to the values estimated for protein interiors. Measurements using these two solvents gave similar transfer free energy values for each of the various hydrophobic amino acids. Such free energy values for transfer from organic solvent to water are negative (e.g. spontaneous) for hydrophilic amino acids and positive (e.g. spontaneous in the opposite direction) for hydrophobic amino acids. While the original work [32] focused on the hydrophobic amino acids, later scales (reviewed in [31]) provided values for both hydrophobic and hydrophilic amino acids. To reflect the balanced importance of both hydrophobic and hydrophilic amino acids as well as to indicate a scale with both types of amino acids, Kyte and Doolittle [31] changed the name of the scale from "hydrophobic" to "hydropathic." They explained their revised name as follows: "Since hydrophilicity and hydrophobicity are no more than two extremes of a spectrum, a term that defines that spectrum would be as useful as either, just as the term light is as useful as violet light or red light. Hydropathy (strong feeling about water) has been chosen for this purpose" [31]. Since the original work of Nozaki and Tanford [32], many hydropathy scales or indices have been developed using a variety of experimental or computational methods to estimate the transfer free energy values [31,33-53].

The ExPASy server [54] alone provides 19 different hydrophopathy scales in ProtScale [55]. Even after normalization, the hydrophobicity value for each amino acid fluctuates by a large amount in the different scales. This raises the possibility that the prediction accuracy of the C-H plot could be improved by using a different hydropathy scale.

Here we used the C-H plot formalism to compare the structure-disorder prediction accuracy when combined with net charge for the 19 hydropathy scales from ExPASy along with the prediction accuracies for other amino acid indices obtained from TOP-IDP [56], FoldUnfold [57], B-value [58], and DisProt [56,59-61]. Next we used the formalism underlying the linear support vector machine [62,63] to develop a new hydropathy scale that further improves prediction of IDPs. As we show by several measures, our new scale, which we first named SVM parameters scale, and later addressed as IDP-Hydropathy scale after showing its high correlation with hydropathy, gives substantially improved predictions as compared to the originally used Kyte-Doolittle scale and also as compared to the best of the tested hydropathy scales. Here we report these comparisons of the various hydropathy scales as well our analysis of their predictions and prediction errors on our set of fully structured and fully disordered proteins. A correlation study between IDP-Hydropathy scale and various clusters with different amino acid properties of Amino Acid index database (AAindex) shows that this new scale is highly correlated with hydropathy [51-53,64,65]. In addition to improved predictions using the C-H plot, we speculate that, given the strong negative correlation between crystallographic disorder and hydropathy [66], our new scale would likely improve disorder prediction for any algorithm that uses hydropathy as one of the inputs.

## Results

### Comparing Hydropathy scale of Kyte-Doolittle (1982) with 18 other hydropathy scales

The C-H plot developed by Uversky et al [3] is a straightforward, simple, fast, yet effective whole protein disorder versus order predictor. FoldIndex is a per residue predictor adapted from the C-H plot, using the same features of charge and hydropathy as the C-H plot[24]. Because of their dependence on intuitive biophysical features and their simplicity, both methods are still heavily used today. However, unlike net charge, which is fairly unambiguous at neutral pH, a variety of hydropathy scales have been developed using quite different methods and assumptions. Thus, the various scales have the potential of being more or less useful, depending on the application.

The hydropathy scale of Kyte-Doolittle (1982) [31] has been used in both the whole protein predictor based on the CH-plot and in the FoldIndex per residue predictor. Therefore, one natural question to ask is, how well do other hydropathy scales perform compared to this particular hydropathy scale? To compare the performances of various hydropathy scales, the 19 different hydropathy scales from ExPASy were tested via C-H plots to predict the structure - disorder status of the proteins in our dataset. The results of this experiment are given in Table 1.

**Table 1:** The Order versus Disorder Prediction Performances of 19 Hydropathy Scales.

Scales	Sens	Spec	Bal. Acc	AUC
Guy	0.70 ± 0.16	0.97 ± 0.02	0.84 ± 0.09	0.90 ± 0.06
Miyazawa	0.70 ± 0.15	0.96 ± 0.02	0.83 ± 0.09	0.90 ± 0.11
Manavalan	0.70 ± 0.15	0.96 ± 0.03	0.83 ± 0.09	0.90 ± 0.07
Sweet	0.69 ± 0.14	0.97 ± 0.02	0.83 ± 0.09	0.91 ± 0.07
Fauchere	0.68 ± 0.13	0.97 ± 0.02	0.83 ± 0.08	0.88 ± 0.07
Rose	0.67 ± 0.17	0.97 ± 0.02	0.82 ± 0.09	0.91 ± 0.06
Black	0.64 ± 0.09	0.97 ± 0.02	0.81 ± 0.06	0.88 ± 0.06
Woods	0.61 ± 0.15	0.97 ± 0.03	0.79 ± 0.09	0.88 ± 0.06
Breese	0.64 ± 0.12	0.95 ± 0.04	0.80 ± 0.08	0.87 ± 0.08
Leo	0.61 ± 0.12	0.96 ± 0.03	0.79 ± 0.08	0.86 ± 0.08
Kyte-Doolittle	0.61 ± 0.16	0.96 ± 0.03	0.79 ± 0.09	0.87 ± 0.10
Roseman	0.56 ± 0.16	0.96 ± 0.02	0.76 ± 0.09	0.86 ± 0.08
Chothia	0.55 ± 0.13	0.96 ± 0.03	0.76 ± 0.08	0.88 ± 0.05
Argos	0.54 ± 0.10	0.97 ± 0.03	0.76 ± 0.06	0.85 ± 0.06
Janin	0.52 ± 0.16	0.96 ± 0.02	0.74 ± 0.09	0.86 ± 0.06
Tanford	0.49 ± 0.14	0.96 ± 0.03	0.73 ± 0.08	0.86 ± 0.09
Eisenberg	0.48 ± 0.19	0.96 ± 0.03	0.72 ± 0.11	0.85 ± 0.05
Welling	0.40 ± 0.14	0.97 ± 0.03	0.69 ± 0.09	0.79 ± 0.07
Wolfenden	0.36 ± 0.11	0.97 ± 0.02	0.67 ± 0.07	0.79 ± 0.06

For equations and explanations, see the Methods section at the end of this manuscript:

Sens: Sensitivity

Spec: Specificity

Bal. Acc: Balanced accuracy (average of sensitivity and specificity)

AUC: Area under the curve

The sensitivity (true positive prediction of disorder, first column in Table 1) and specificity (true positive prediction of order, second column in Table 1) are averaged to give the balanced accuracy (third column in Table 1). As shown in Table 1, many other hydropathy scales from ExPASy achieved a higher balanced accuracy when compared to the Kyte-Doolittle hydropathy scale. Another commonly used measure of predictor quality is the area under the receiver operator characteristic curve, commonly abbreviated as AUC. Just as for the balanced accuracy, the AUC metric indicates that the Kyte-Doolittle scale is far from the best with regard to classification of ordered and disordered proteins (Table 1, column 4).

While the balanced accuracy and AUC values give easy-to-interpret measures of predictor performance and so are widely used, these metrics have deficiencies for predictors trained on unbalanced datasets. For such imbalanced datasets, over-predicting the minority examples leads to a false indication of improvement because such over-prediction leads to only small errors in the majority examples [67] (see Methods for more discussion). As a result, we further evaluated the results using metrics designed to evaluate predictors trained on imbalanced data (Table 2), including the F-score (Table 2 column 1), Matthews Correlation Coefficient (MCC, Table 2, column 2), Positive Predictive Values (PPV, Table 2, column 3), and Negative Predictive Values (NPV, Table 2, column 4, see Methods for more discussion of these metrics). The F-score and MCC values both provide a good summary of a predictor's overall performance. The PPVs and NPVs indicate whether the algorithm over-predicts the indicated class.

**Table 2:** The Order versus Disorder Prediction Performances of 19 Hydropathy Scales Measured by Other Metrics.

Scales	F	MCC	PPV	NPV
Guy	0.75 ± 0.12	0.71 ± 0.13	0.82 ± 0.10	0.94 ± 0.03
Miyazawa	0.74 ± 0.11	0.70 ± 0.12	0.80 ± 0.10	0.94 ± 0.03
Manavalan	0.74 ± 0.11	0.70 ± 0.12	0.80 ± 0.10	0.94 ± 0.03
Sweet	0.74 ± 0.08	0.71 ± 0.08	0.83 ± 0.11	0.94 ± 0.03
Fauchere	0.74 ± 0.08	0.70 ± 0.09	0.83 ± 0.12	0.94 ± 0.02
Rose	0.73 ± 0.12	0.70 ± 0.13	0.82 ± 0.09	0.94 ± 0.03
Black	0.71 ± 0.05	0.67 ± 0.06	0.81 ± 0.12	0.93 ± 0.02
Woods	0.68 ± 0.12	0.64 ± 0.13	0.78 ± 0.12	0.93 ± 0.03
Breese	0.68 ± 0.11	0.63 ± 0.13	0.75 ± 0.15	0.93 ± 0.02
Leo	0.68 ± 0.10	0.64 ± 0.12	0.79 ± 0.15	0.93 ± 0.02
Kyte-Doolittle	0.67 ± 0.13	0.63 ± 0.14	0.78 ± 0.14	0.93 ± 0.03
Roseman	0.64 ± 0.15	0.59 ± 0.17	0.75 ± 0.15	0.92 ± 0.03
Chothia	0.63 ± 0.11	0.59 ± 0.13	0.77 ± 0.15	0.92 ± 0.03
Argos	0.63 ± 0.09	0.59 ± 0.10	0.78 ± 0.13	0.92 ± 0.02
Janin	0.59 ± 0.14	0.55 ± 0.12	0.74 ± 0.11	0.91 ± 0.03
Tanford	0.57 ± 0.14	0.53 ± 0.14	0.72 ± 0.15	0.91 ± 0.02
Eisenberg	0.56 ± 0.16	0.53 ± 0.18	0.74 ± 0.20	0.91 ± 0.03
Welling	0.50 ± 0.15	0.48 ± 0.13	0.78 ± 0.19	0.89 ± 0.02
Wolfenden	0.46 ± 0.11	0.43 ± 0.13	0.69 ± 0.15	0.89 ± 0.02

For equations and explanations, see the Methods section at the end of this manuscript:

F: the F1 score

MCC: Matthew Correlation Coefficient

PPV: Positive Predictive Values

NPV: Negative Predictive Values

Predictor training for the data in Tables 1 and 2 were carried out so as to optimize the F-score (Table 2, column 1). The results show that, just as for the balanced accuracy and AUC metrics (Table 1), the hydropathy scale of Kyte-Doolittle (1982) is only average, giving 0.67 for the F-score, ranking in the middle of the 19 hydropathy scales. The Guy (1985) hydropathy scale gives the highest F-score, a value of 0.75, which is a 12% improvement compared to the hydropathy scale of Kyte-Doolittle (1982). Also, the use of the Guy (1985) scale maintains a PPV score of 0.82, suggesting that the gain in its sensitivity (Table 1) is not from an overly large increase in its false positive rate. Clearly the Guy (1985) hydropathy scale gives improved performance compared to that of Kyte-Doolittle (1982) when used with net charge to classify structured and disordered proteins via the C-H plot. Note that, because predictor training was carried out so as to optimize the F-score, sensitivity (correct predictions of disorder) and specificity (correct predictions of order) give values that are very different from each other.

### Finding a hydropathy scale for improved prediction of IDPs

Since disorder prediction based on C-H plot can be significantly improved by simply adopting a different hydropathy scale, it seems reasonable to ask whether another hydropathy scale can be found or developed that further improves the performance of the C-H plot.

#### Use of Linear SVMs to find a hydropathy scale giving an improved classification

To find a hydropathy scale that gives an improved order-disorder classification via the C-H plot methodology, we adopted a linear support vector machine (SVM) [68] for this purpose. SVMs represent a new generation of learning systems based on recent advances in statistical learning theory [62,63]. The aim in training a linear SVM is to find the separating hyperplane with the largest margin; the expectation is that the larger the margin, the better the generalization of the classifier. Typically, the weights that are found as giving the best performance are viewed as arbitrary parameters. However, in this particular instance, the SVM weight given to each amino acid, when appropriately normalized, corresponds to its hydropathy value.

Given the above, we rephrase the question of finding the optimal scale by viewing sets of protein sequences/windows as an *n *by 21 matrix (Eq. 1). The *n *rows represent n protein sequences/windows, and 21 columns are comprised of 20 normalized amino acid compositions and normalized net charge. For sequence window *i*, *Comp_ij _*is its *j*'s amino acid composition, and *C_i _*is its normalized net charge, calculated as (Eq. 2). We represent the disorder/order status of *i*th protein sequence/window as *Y_i _*(-1 or 1), thus giving:

(1) $$\left[\begin{array}{c} Y1\\ Y2\\ \begin{array}{c} \vdots \\ \vdots \\ Yn \end{array} \end{array}\right]=\left[\begin{array}{ccc} Comp_{11} & Comp_{12} & \begin{array}{ccc} \ldots & Comp_{20} & C1 \end{array}\\ Comp_{21} & Comp_{22} & \begin{array}{ccc} \ldots & Comp_{20} & C2 \end{array}\\ \begin{array}{c} Comp_{31}\\ \ldots \\ Comp_{n1} \end{array} & \begin{array}{c} Comp_{32}\\ \ldots \\ Comp_{n2} \end{array} & \begin{array}{ccc} \begin{array}{c} \ldots \\ \ddots \\ \ldots \end{array} & \begin{array}{c} Comp_{20}\\ \ldots \\ Comp_{20} \end{array} & \begin{array}{c} C3\\ \ldots \\ Cn \end{array} \end{array} \end{array}\right]*\left[\begin{array}{c} w1\\ w2\\ \begin{array}{c} \vdots \\ \vdots \\ \begin{array}{c} w20\\ w21 \end{array} \end{array} \end{array}\right]+b,$$

(2) $$whereC_{i}=Comp_{iArg}+Comp_{iLys}-Comp_{iGlu}-Comp_{iAsp}.$$

Note that, to conform to the energy transferring convention set by Kyte & Doolittle, disordered examples are assigned with Y values of -1, such that a negative weight will be disorder promoting. Then, the linear SVM is employed here to find a 21 by 1 weight vector *w*, such that *wM*+*b *(bias) is closest to Y (Eq. 1). We then adopted the w1 to w20 values as 'SVM parameters scale'. As shown later, this SVM parameters scale is highly correlated with amino acid hydropathy, and then we change its name into 'IDP-Hydropathy scale'. For now, we address it as SVM parameters scale. Because the first published C-H plot by Uversky normalized the Kyte-Doolittle scale to the interval of 0 to +1, when we were plotting the C-H plot later, we normalized our scale to the interval of 0 and +1 for easier comparison among each scale.

We previously showed that amino acid compositions associated with disordered segments exhibit changes that depend on segment length [69] and that construction of length-dependent predictors gives improved performance [17]. To minimize such length-dependent variation, we tested whether use of uniform-sized segments of protein during training would improve the subsequent classifiers based on the C-H plot. We found this to be the case. We tried a wide range of window sizes, and based on these results we chose a value of 41 residues. The reasons for choosing this size are that, first, this window size yields good prediction accuracy, and, second, this window size is smaller than almost all of the smallest currently known self-folding domains.

The scale was constructed from the weight vector found by the SVM. To be consistent with the original C-H plot paper, and with previous hydropathy scale test results, this scale is applied and tested over the entire protein sequences. A 10-fold cross validation was used here, and was reiterated 5 times in this method. We also tested a genetic algorithm [70] and an elastic net [71] (i.e., a penalized logistic regression classifier) as alternatives for the generation of the best hydropathy scale for the order/disorder classification via the C-H plot. Both of these approaches give scales with prediction performance values similar to those obtained by the SVM methodology. We chose to present the SVM approach because of its greater simplicity and elegance compared to the other methods.

The new scale developed using the SVM formalism shows an improved performance compared to the tested 19 scales, namely: 0.84 F-score, 0.81 sensitivity, 0.98 specificity, 0.90 balanced accuracy, 0.94 AUC, and 0.89 PPV. We named this scale "SVM parameters scale" for now, and its values for the 20 amino acids are given in Table 3. Also shown in Table 3 are the Kyte-Doolittle and Guy hydropathy scales so their differences can be compared. A more in-depth comparison of these three scales is discussed later.

**Table 3:** A comparison of 3 hydropathy scales.

IDP-Hydropathy scale										
Residue	W	Y	I	F	C	L	V	M	N	T
**Hydropathy Score**	10.66	6.64	6.19	5.79	5.62	5.17	4.64	2.49	2.06	1.22
**Residue**	**A**	**R**	**G**	**D**	**Q**	**S**	**H**	**E**	**K**	**P**

**Hydropathy Score**	0.91	0.07	0.02	-0.48	-1.23	-1.84	2.18	-2.20	-2.43	-3.89
**Guy scale**										
**Residue**	**W**	**Y**	**I**	**F**	**C**	**L**	**V**	**M**	**N**	**T**

**Hydropathy Score**	-0.51	-0.21	-1.13	-2.12	-1.42	-1.18	-1.27	-1.59	0.48	0.07
**Residue**	**A**	**R**	**G**	**D**	**Q**	**S**	**H**	**E**	**K**	**P**

**Hydropathy Score**	0.10	1.91	0.33	0.78	0.83	0.52	-0.50	0.95	1.40	0.73
**Kyte-Doolittle scale**										
**Residue**	**W**	**Y**	**I**	**F**	**C**	**L**	**V**	**M**	**N**	**T**

**Hydropathy Score**	-0.90	-1.30	4.50	2.80	2.50	3.80	4.20	1.90	-3.5	-0.70
**Residue**	**A**	**R**	**G**	**D**	**Q**	**S**	**H**	**E**	**K**	**P**

**Hydropathy Score**	1.80	-4.50	-0.40	-3.50	-3.50	-0.80	-3.20	-3.50	-3.90	-1.60

### Comparing C-H Plots for three scales

**Figure 1:**
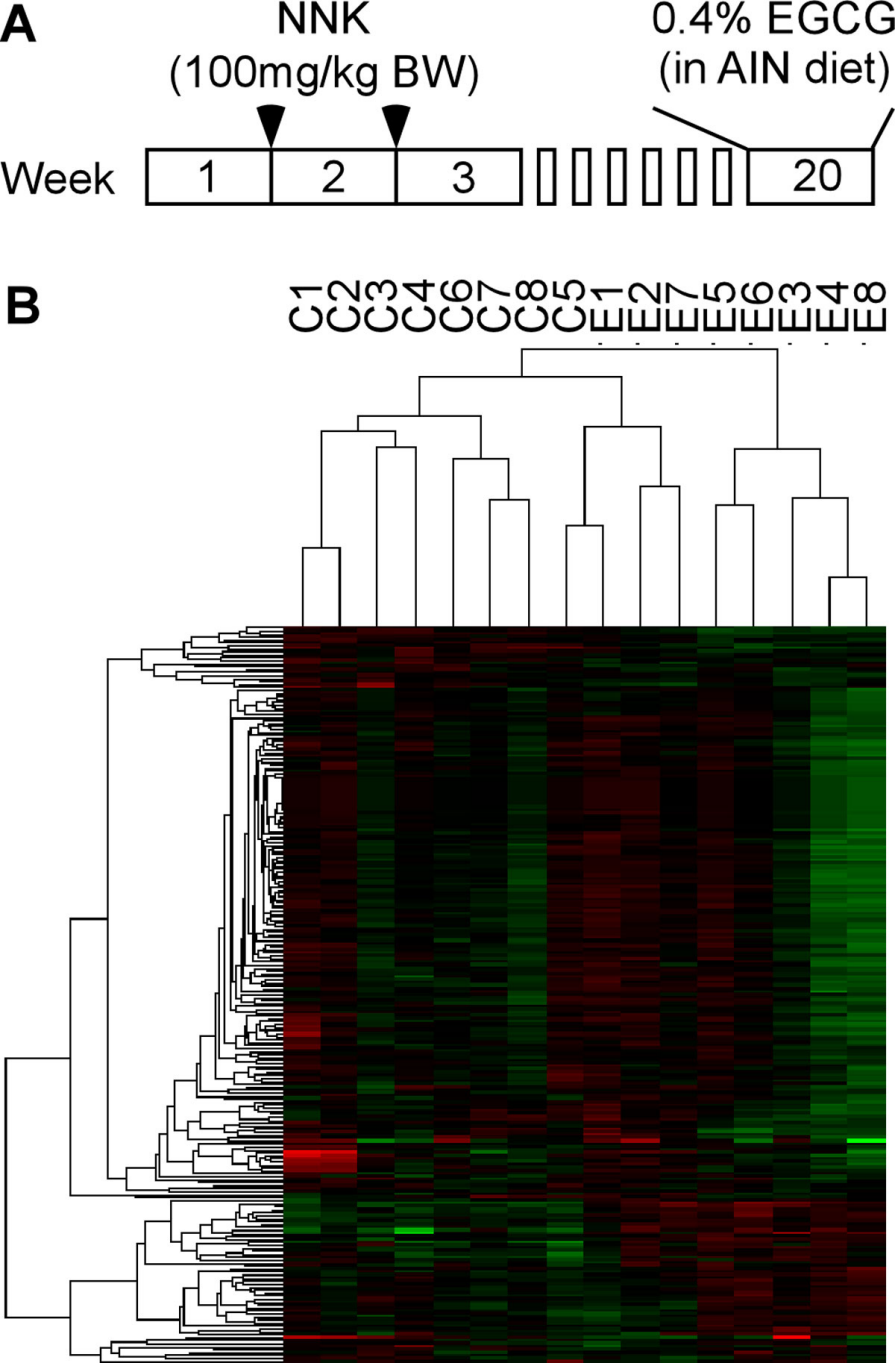
**Charge-Hydropathy plots**. In (A) the IDP-Hydropathy scale was used, in (B) the Guy (1985) Hydropathy scale was used, and in (C) the Kyte-Doolittle (1981) hydropathy scale was used. Red circles indicate disordered proteins, blue circles indicate structured proteins. For these plots, each scale was normalized to be in the interval of 0 to 1. The Guy's scale is multiplied by -1 prior to normalization to conform to the energy rule set by Kyte-Doolittle scale. In (A) the function describing the boundary is: <charge> = 3.31 <hydropathy> -0.97. In (B) the function describing the boundary is: <charge> = 2.32 <hydropathy> -0.93. In (C), the function describing the boundary is: <charge> = 1.35 <hydropathy> -0.49.

The C-H plots generated using scale SVM parameters scale, Kyte-Doolittle hydropathy scale, and Guy hydropathy scale for whole protein prediction are shown in Figure 1. Figure 1A, which is derived by SVM parameters scale, shows many fewer misclassified disordered proteins on the ordered side, compared to Figure 1B and 1C.

### SVM parameters scale is highly correlated with other amino acid hydropathy scales

Since SVM parameters scale is derived via computation, and focused on maximizing prediction accuracy rather than being based on experimentally measured physical attributes, another question to ask is if this scale is truly a hydropathy scale or if it contains input from other amino acid properties. One way to test this possibility is to study how this scale correlates with non-hydropathy and hydropathy scales.

To obtain sets of amino acid indices grouped according to their properties, we referred to the AAindex cluster analysis by Tomii et al [65]. AAindex is a database of numerical indices for various amino acids physicochemical and biochemical properties [51-53]. Tomii et al clustered the AAindex into 6 clusters according to the absolute value of correlation coefficient (|*r*|) between pairs of amino acid indices. These 6 clusters are, α and turn propensities (A), β propensity (B), Composition (C), Hydropathy (H), Physicochemical properties (P), and Other properties (O).

**Figure 2:**
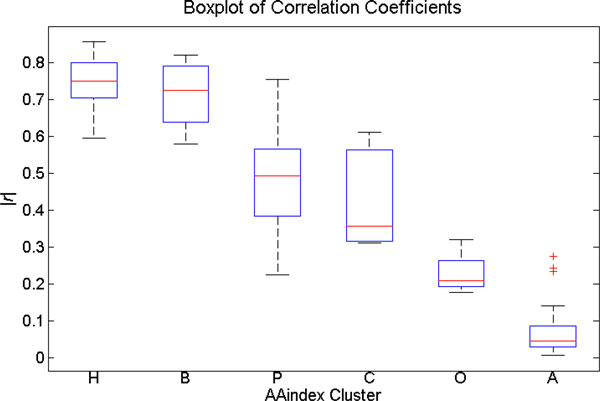
**Correlation coefficients between IDP-Hydropathy and AAindex clusters**. H: Hydrophobicity cluster B: β propensity cluster P: Physicochemical properties cluster C: Composition cluster O: Other properties cluster A: α and turn propensities

The correlation coefficients of the SVM parameters scale and each amino acid scales from all 6 clusters are shown in Figure 2 and Table 4. Ordered by averaged |*r*| values, the SVM parameters scale is shown to be most correlated with the Hydropathy cluster with an average |*r*| of 0.73. Interestingly, SVM parameters scale is also very closely correlated with the β propensity cluster with an average |*r*| of 0.72. Note that β sheets have a high occurrence of aromatic residues such as Tyr, Phe and Trp, and such residues tend to be strongly depleted in disordered proteins, thus resulting in a high value for |*r*|. Other non-hydropathy AAindex clusters are much less correlated with our newly developed scale. This suggests that the SVM parameters scale is indeed strongly related to other hydropathy scales with little input from other properties. We thus refer to this scale as the IDP-Hydropathy scale from now on.

**Table 4:** Mean, median, standard deviation, max, and min of |*r*| and AAindex in each cluster.

Cluster	Mean	Median	Std	Max	Min
H	0.75	0.75	0.07	0.86	0.60
B	0.72	0.72	0.08	0.82	0.58
P	0.49	0.49	0.16	0.76	0.23
C	0.43	0.36	0.13	0.61	0.31
O	0.23	0.21	0.06	0.32	0.18
A	0.08	0.05	0.08	0.28	0.01

H: Hydropathy cluster

B: β propensity cluster

P: Physicochemical properties cluster

C: Composition cluster

O: Other properties cluster

A: α and turn propensities cluster

### Comparing the IDP-Hydropathy scale with the Doolittle and Guy hydropathy scales

**Figure 3:**
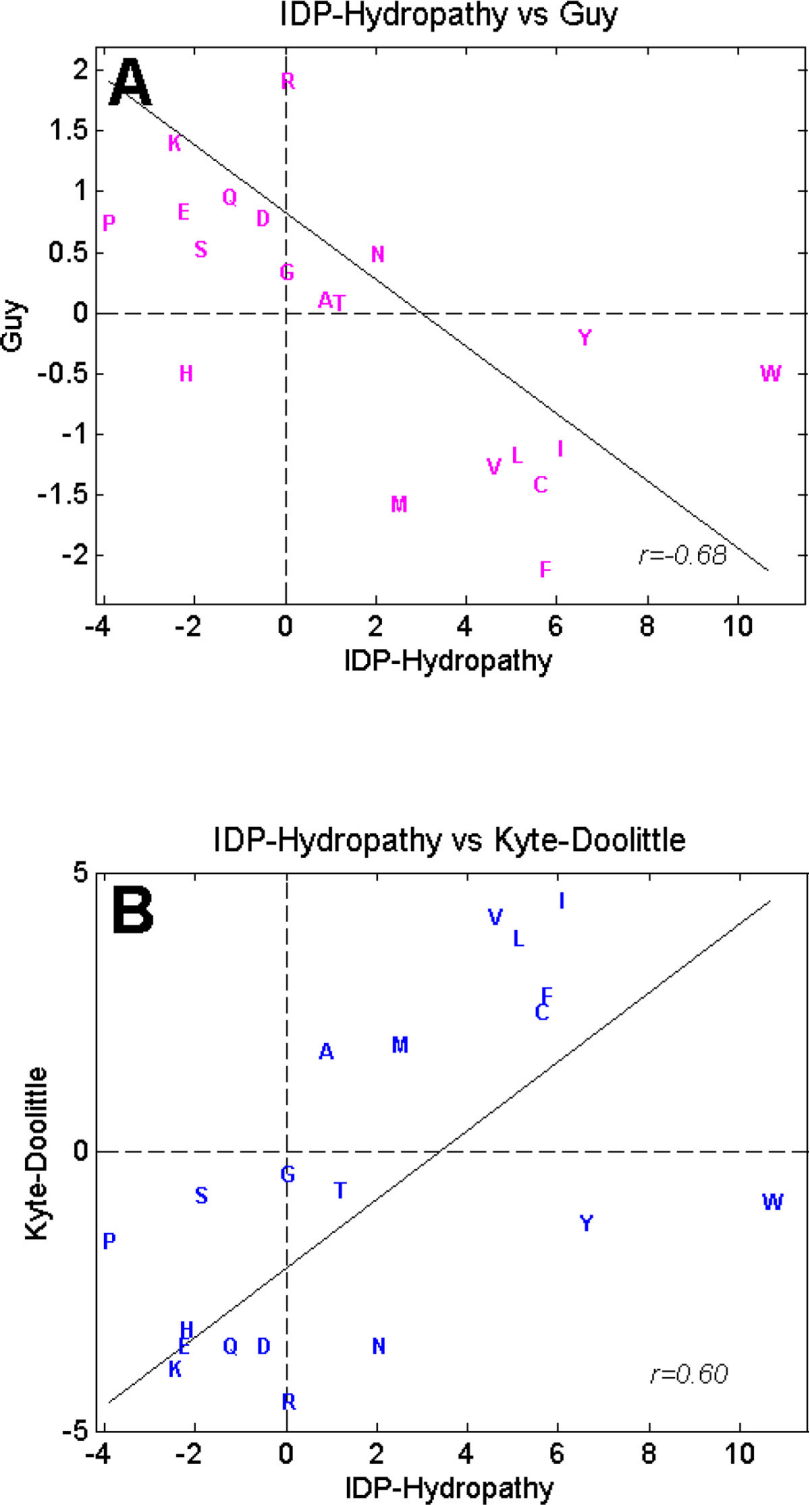
**Comparing IDP-Hydropathy scale against Guy's scale (A) and Kyte-Doolittle's scale (B)**. Each letter is the one letter code for an amino acid. Note that in Guy's scale (A), the measurement for free energy transfer adopted the opposite theme as compared to the Kyte-Doolittle scale. In Guy's scale, a positive value indicates hydrophilic, while in Kyte-Doolittle scale and IDP-Hydropathy, a positive value indicates hydrophobic. The *r *value is the correlation coefficient of the 2 scales compared.

A detailed comparison of IDP-Hydropathy scale to other hydropathy scales provides further understanding of this new scale. In Figure 3, the hydropathy scores of each amino acid residue in Guy (Figure 3A) and Kyte-Doolittle (Figure 3B) scales are plotted against the scores in IDP-Hydropathy scale. If the scores from the two scales compared are equal, that amino acid residue would appear on the solid line given in each plot (Figure 3AB). Keep in mind that Kyte-Doolittle scale was calculated with a minus sign in front of the energy transfer function, while Guy scale was not [31,33]. Thus, the hydrophobic residues have positive values for Kyte-Doolittle scale (Figure 3B, quadrant 1 and 4) but negative values (Figure 3A, quadrant 2 and 3) for the Guy scale. The IDP-Hydropathy scale is designed to follow the rule set by Kyte-Doolittle scale, in which hydrophobic residues are positive (Figure 3A and 3B, quadrant 1 and 2) and hydrophilic residues are negative (Figure 3A and 3B, quadrant 3 and 4). From these plots and the data in Table 3 (above), the values for the following amino acids show step-wise changes in the same direction thus correlating with the increased accuracy in the order/disorder classification, where the indicated amino acid is followed by the hydropathy values in order from Kyte-Doolittle-, to Guy, to IDP-Hydropathy:; W, - 0.90, - 0.51, + 10.66; Y, -1.3, - 0.21, + 6.64; A, + 1.80, + 0.10, + 0.91; G, - 0.40, + 0.33, + 0.02; and P, - 1.60, + 0.73, - 3.89. In both of Figure 3A and 3B, W and Y are located in quadrant 2, indicating that they are hydrophobic in Guy and IDP scale, but hydrophilic in Kyte-Doolittle scale. In fact, Kyte-Doolittle[31] suggested that W and Y are slightly hydrophilic due to their hydrogen bonding potential, whereas most hydropathy scales classify these amino acids as hydrophobic. The IDP-Hydropathy ranks W as the most hydrophobic (+ 10.66) of all, despite its hydrogen bonding potential. Interestingly, Kyte-Doolittle ranks A as quite hydrophobic (+ 1.80), while both Guy and IDP-Hydropathy rank this amino acid as somewhat hydrophilic. G is ranked as hydrophilic in all three scales with larger values as the classification accuracy improves. Finally, despite its hydrophobic side chain, proline is indicated to be hydrophilic by all three scales, and being the most hydrophilic residue of all (e.g. a value of - 3.89) in the IDP-Hydropathy scale. This counter-intuitive result arises from the lack of NH groups on the proline peptide bonds, leading to hydrogen bond acceptors from the carbonyl oxygen but no corresponding donors. This donor/acceptor imbalance makes it very costly in terms of energy to bury proline's backbone atoms. Indeed, because of this imbalance, proline is the most soluble of all the amino acids at neutral pH [72], and polyproline is far more soluble than polyleucince, polyalanine and even polyglycine [73].

Thus, when the backbone is taken into account, proline becomes a very hydrophilic amino acid [74].

### Hydropathy versus other scales related to protein folding

The C-H plot assumes the biophysical model that net charge repulsion favors the unfolded state while hydropathy favors the folded state. What if other factors also contribute significantly to protein folding? Thus, replacing the hydropathy scale in the C-H formalism with another scale that differentiates between structured and disordered proteins has the potential of improving the order/disorder classification.

Several amino acid scales have been developed that are related to whether a protein folds or folds tightly. These include the fractional differences in the amino acids found in structured proteins compared with those found in the disordered proteins and regions in the DisProt database [59,60] as described in Campen et al [56]. and herein called DisProt, a scale based on improved classification of ordered and disordered regions of proteins called TopIDP [56], a scale based on side chain packing capacity called FoldUnfold [57] and a scale based on the B-factor values for the different residues averaged over multiple protein structures [58] herein called B-value. Thus, using each of these scales along with net charge via the C-H plot formalism might give better classification than using scales based on hydropathy alone. Table 5 gives the results of replacing the hydropathy scale with each of the four disorder propensity scales along with the results of IDP-Hydropathy and the Guy and Doolittle scales for comparison. In this comparison, IDP-Hydropathy again ranks on as the best, followed by DisProt, Top-IDP, Fold-Unfold, Guy, B-value, and Doolittle. Thus, when combined with net charge, IDP-Hydropathy is a better indicator of whether a protein is structured as compared to these alternative measures.

**Table 5:** IDP-Hydropathy scale performance compared to 4 disorder propensity scales, DisProt, TopIDP, FoldUnfold, and B-value.

Method	Sens	Spec	Bal. acc	AUC	F	MCC	PPV	NPV
**IDP-Hydro**	0.81 ± 0.11	0.98 ± 0.02	0.90 ± 0.07	0.94 ± 0.05	0.84 ± 0.08	0.82 ± 0.09	0.89 ± 0.09	0.96 ± 0.02
**DisProt**	0.77 ± 0.12	0.97 ± 0.04	0.87 ± 0.08	0.94 ± 0.06	0.80 ± 0.08	0.77 ± 0.10	0.85 ± 0.14	0.96 ± 0.02
**TopIDP**	0.76 ± 0.11	0.97 ± 0.02	0.87 ± 0.07	0.93 ± 0.04	0.79 ± 0.06	0.76 ± 0.06	0.84 ± 0.07	0.96 ± 0.02
**FoldUnfold**	0.72 ± 0.12	0.97 ± 0.02	0.85 ± 0.07	0.91 ± 0.07	0.77 ± 0.10	0.73 ± 0.11	0.82 ± 0.11	0.95 ± 0.02
**Guy**	0.70 ± 0.16	0.97 ± 0.02	0.84 ± 0.09	0.90 ± 0.06	0.75 ± 0.12	0.71 ± 0.13	0.82 ± 0.10	0.94 ± 0.03
**B-value**	0.67 ± 0.14	0.98 ± 0.02	0.83 ± 0.08	0.91 ± 0.07	0.74 ± 0.11	0.71 ± 0.12	0.85 ± 0.10	0.94 ± 0.02
**Kyte-Doolittle**	0.61 ± 0.16	0.96 ± 0.03	0.79 ± 0.09	0.87 ± 0.10	0.67 ± 0.13	0.63 ± 0.14	0.78 ± 0.14	0.93 ± 0.03

The accuracy metrics for Guy and Kyte-Doolittle hydropathy scales are also presented as references.

For equations and explanations, see the Methods section at the end of this manuscript:

Sens: Sensitivity

Spec: Specificity

Bal. Acc: Balanced accuracy (average of sensitivity and specificity)

AUC: Area under the curve

F: the F1 score

MCC: Matthew Correlation Coefficient

PPV: Positive Predictive Values

NPV: Negative Predictive Values

### Disorder is harder to predict

One interesting observation here is that across all tested hydropathy scales, including the IDP-Hydropathy, the specificity is high (>0.96) for all predictors, while the sensitivity is quite low compared to specificity. These scales were developed, not by attempting to obtained equal-accuracy predictions on structure and disorder, but rather by optimizing the F-value, which was developed to deal with imbalanced data [57]. Of the 19 ExPAsy hydropathy scales, the highest sensitivity is only 0.70 (Table 1). IDP-Hydropathy also has a relatively large gap between its sensitivity (0.81) and specificity (0.98). The straightforward interpretation of these results is simply that disorder is harder to predict than structure. We hypothesize that this results from the frequent occurrence of segments having a high tendency to form structure within experimentally characterized disordered proteins and regions.

This hypothesis is supported by running per residue predictors, PONDR^® ^VLXT [16] and VSL2 [17] on our whole disordered/structured protein dataset. Fractions of predicted disorder and order over the entire dataset by each predictor are displayed in Table 6. The PONDR^® ^VLXT algorithm predicts residue disorder tendencies within a narrow window, and is built to be very sensitive to local features in protein sequences. PONDR^® ^VSL2, on the other hand, uses a longer window, and so its prediction is smoother with less focus on local changes. In Table 6, on average, PONDR^® ^VLXT predicts only 58% disordered residues within an entirely disordered protein, while it predicts 78% structured residues for the sequence of wholly structured protein. The PONDR^® ^VSL2 prediction results are quite different. VSL2 has a comparable amount of predicted disorder residues within disordered protein as predicted structure in a structured protein. This suggests that indeed, there are many short segments with potential for structure-formation within regions within a disordered protein.

**Table 6:** VLXT and VSL2 per residue prediction over our entirely disordered/structured dataset.

		**Predicted**			
		
		***VLXT***		***VSL2***	
		
		**Disorder**	**Structure**	**Disorder**	**Structure**
		
**Dataset**	Disordered	58%	~	78%	~
	Structured	~	78%	~	74%

The entries are fraction of residues that are predicted disordered/structured over the whole disordered/structured dataset. For simplicity, only the diagonal entries for each predictor are shown.

## Discussion

Here we show that the performance of C-H plot can be improved significantly by introducing a new hydropathy scale. This new IDP-Hydropathy scale boosts the predictor's F-score from an original value of 0.67 to the 25% higher value of 0.84. This new scale also performs considerably better than four existing disorder propensity-based scales. A correlation study between this scale and clusters of different amino acid indices shows that this scale is indeed highly associated with amino acid hydropathy.

In all of our tested scales, including IDP-Hydropathy, disorder prediction accuracy is much lower than the order prediction accuracy. We hypothesize that this results from the existence of many small regions with increased order propensity that are located inside larger disordered regions. Despite of these short structure-prone elements, these regions are still experimentally shown to be mostly disordered. These regions with increased order propensity are often found to be functional domains within the disordered proteins. Molecular recognition features (MoRFs)[75,76] that bind to specific protein or nucleic acid partners are one type of disorder-based functional regions. When not bound to a partner, such MoRF segments remain disordered and flexible. Upon binding, they typically become structured, adopting ordered conformations that depend on the templates provided by the binding partners. Their flexibility in the unbound state allows them change their shape as needed to fit onto the surfaces of different and distinct partners [5,75,77,78].

This new scale, IDP-Hydropathy derived from entirely disordered and structured proteins, is a very handy tool because of its simplicity and prediction power. This new scale should improve other disorder predictors that use hydropathy as one of the input features. We are looking forward to the incorporation of this new scale into a per-residue predictor based on these same principles.

## Conclusions

The original hydrophobicity scale of Nozaki and Tanford[32] was developed with the purpose of understanding the relative importance of different amino acids to protein folding. The IDP-Hydropathy scale developed here is based on sets of sequences that fold into 3D structure as compared to collections of sequence that don't fold, using the C-H plot as the classifier. Thus, to a very significant degree, IDP-Hydropathy fulfills the intent of the original scale by providing a measure of how the various amino acids contribute to protein folding by means of their hydropathy values.

## Methods

### Dataset

Two sets of proteins were used in this study [19,79]: experimentally verified entirely disordered proteins and experimentally verified completely structured or ordered proteins. Entirely disordered proteins were taken from Disprot 6.0 [59,60]. These proteins were filtered such that only those proteins with their entire sequences being disordered were retained. Our fully disordered protein dataset contains 109 disordered sequences with 22,614 amino acid residues. The set of fully structured (ordered) proteins consisting only of single-chain and non-membrane proteins was assembled from the Protein Data Bank (PDB)[80]http://www.rcsb.org/pdb/. Only structures determined by X-ray crystallography and characterized by unit cells with primitive space groups were kept in our dataset. Structures with ligands, disulfide bonds, or missing residues were also removed. Then a BLASTCLUST [81] analysis was performed to cluster proteins into subsets, with all members of each subset having at least 25% sequence identity with another subset member and having less than 25% sequence identity with any member of any other subset. The longest sequence in each cluster was selected to construct the fully ordered protein set. This set of experimentally determined structured proteins contains 563 fully structured protein sequences with 113,895 amino acid residues.

### Training method

In the current dataset, disordered proteins are outnumbered and under-represented. To develop a good predictor in the scenario of unbalanced dataset, we tried several popular methods [67]. Both under-sampling structured proteins, and oversampling disordered proteins [82-84] were implemented separately to achieve a balanced disorder/order dataset. Synthesizing new data for the disordered class was also carried out to obtain more disordered samples [85,86]. We found that in this study, all of these methods gave similar results. The approach of adding weights to the SVM cost function [62,67,71] so that a greater penalty occurs when a disordered protein is misclassified, achieves results similar to the sampling methods above while being much simpler to implement compared to under- or oversampling. Therefore, for simplicity, here we only used the approach of using a weighted cost function.

The entire dataset is divided into 10 subsets for 10 fold cross-validation. For each subset, the whole protein sequences are further chopped into small windows of length 41 amino acids. The above two processes are iterated until each subset has approximately the same number of small protein windows. The trained parameters from each training set are averaged to obtain the final IDP-Hydropathy scale. In each fold of cross-validation, the windows are reassembled to whole protein to derive the boundary parameters for whole protein disorder prediction. The final parameters are also an average of all 10 folds.

### Dealing with unbalanced data

#### Assessment metrics

Our dataset of disordered/structured proteins is highly imbalanced with 16% disordered and 83.8% structured based on numbers of chains or 17% disordered and 83% structured based on numbers of amino acid residues. Accuracy, defined as the proportion of correctly classified samples in the population (Eq. 3), is not a good measurement when the number of one class dominates [67]. In fact, simply predicting every case as structured would yield accuracy close to 0.84. A better approach is to average the correct prediction of order and the correct prediction of disorder, called the balanced accuracy and calculated as follows: first, estimate the value for the correct prediction of disorder, called sensitivity (Eq. 4), and the value for the correct prediction of structure, called specificity (Eq. 5), then average the values for sensitivity and specificity[67] (Eq. 6):

(3) $$Acc=\frac{TP+TN}{TP+TN+FP+FN},$$

where Acc = accuracy, TP = true positive predictions, TN = true negative predictions, FP = false positive predictions, and FN = false negative predictions,

(4) $$Sensitivity\left(Recall\right)=\frac{TP}{TP+FN},$$

(5) $$Specificity=\frac{TN}{TN+FP},$$

(6) $$BalancedAcc=\frac{Sensitivity+Specficity}{2}.$$

The usefulness of the balanced accuracy metric is undermined by the high fraction of structured residues in the training set. That is, predicting more disordered residues rewards sensitivity much more than the penalty in specificity, so this imbalance encourages overpredicting disorder [25,26,67]. To further help with the analysis of prediction on imbalanced data, the positive predictive value (PPV) metric was introduced[87-89]. PPV, also called "precision", is calculated as the fraction of correctly predicted disorder versus all the predicted disorder (Eq. 7):

(7) $$PPV\left(Precision\right)=\frac{TP}{TP+FP}.$$

Overpredicting disorder will result in low PPV, whereas a high PPV value indicates that a high proportion of the predicted disorder is indeed actual disorder. Combing PPV with sensitivity (also known as recall) as indicated (Eq.8) yields the F-score, which is an effective representation of the predictive power in imbalanced dataset[90]:

(8) $$F=2\cdot \frac{precision\cdot recall}{precision+recall}.$$

The F-score values range from 0 to 1, and because of the product of precision and sensitivity in the numerator, a high F-score usually means a high score for both PPV and sensitivity, or recall.

The Matthews correlation coefficient (MCC) is another very commonly used and effective metric for imbalanced datasets[26,91] (Eq. 9):

(9) $$MCC=\frac{TP\cdot TN-FP\cdot FN}{\sqrt{\left(TP+FP\right)\left(TP+FN\right)\left(TN+FP\right)\left(TN+FN\right)}}.$$

The MCC has been observed to be highly correlated with the F-score for disorder prediction in Critical Assessment of protein Structure Prediction 9 (CASP9)[26].

In contrast to PPV, negative predictive value (NPV) measures the correctly predicted structured proteins over all of the predicted structured proteins[87] (Eq. 10):

(10) $$NPV=\frac{TN}{TN+FN}.$$

A Receiver Operating Characteristic (ROC) curve is a plot of sensitivity versus specificity[92]. The area under the curve (AUC) is another often used metric for judging predictive power of an algorithm.

Given all of the above, we estimated F-score, MCC, sensitivity, specificity, AUC, PPV, and NPV as the metrics to assess the quality of the predictions that were made on the unbalanced dataset used herein. Sensitivity, specificity and AUC are informative about the correctly predicted disorder and structure of one class. PPV and NPV reveal whether the algorithm is overpredicting disorder or structure. In the end, the F-score and MCC give an overall estimate of the quality of the predictions.

### Correlation study

The absolute value of Pearson product-moment correlation coefficient [93], r, was calculated between IDP-Hydropathy scale and shaded indices from AAindex clusters. For each *scale *from AAIndex, the correlation of it with IDP-Hydropathy scale is calculated as in Equation 11, where *IDP_i _*is the score for *i*th amino acid in IDP-Hydropathy scale, *Scale_i _*is the score for *i*th amino acid in that AAIndex. $\bar{IDP}$ and $\bar{Scale}$ stands for the mean value of the two scales:

(11) $$r=\frac{\sum_{i=1}^{20}\left(IDP_{i}-\bar{IDP}\right)\left(Scale_{i}-\bar{Scale}\right)}{\sqrt{\sum_{i=1}^{20}{\left(IDP_{i}-\bar{IDP}\right)}^{2}}\cdot \sqrt{\sum_{i=1}^{20}{\left(Scale_{i}-\bar{Scale}\right)}^{2}}}.$$

### Benchmarking

The IDP-Hydropathy scale was derived from windows of proteins. Since entire protein sequences are applied to the original C-H plot by Uversky et al, for consistency, the benchmarking of IDP-Hydropathy scale and other scales was carried out over the entire protein sequences. The normalized composition and net charge were calculated as before. Then we obtained the 'hydropathy score' for each protein by multiplying the composition matrix and the column vector of the scale. Therefore, 2 attributes are calculated for each amino acid sequences, the 'hydropathy score' and the net charge. A linear SVM classifier was then applied to predict disorder/structure proteins.

For entire protein prediction of per-residue predictors, PONDR-FIT, VSL2, VLXT, VL3, IUPred, the average of their scores are used.

### Charge-Hydropathy plots

C-H plots were generated using our dataset with the following scales: IDP-Hydropathy, the Guy scale [33], and the Kyte-Doolitte (1982) scale [31]. The normalized net charge was calculated as previously: the absolute value of [(Arginine + Lysine) - (Glutamate + Aspartate)]/Protein Length. Then the normalized hydropathy was calculated using the indicated scales. Note that to be consistent with the original C-H plot [3], the various hydropathy scales were renormalized so as to cover the range between 0 and +1 rather than -1 to +1 as we use elsewhere herein. The linear SVM method implemented by LIBLINEAR library[68] was then applied to calculate the boundary in MATLAB (MATLAB 2012a. Natick, Massachusetts: The MathWorks Inc., 2012).

## Competing interests

The authors declare that they have no competing interests.

## Authors' contributions

FH, CO, SL, XL, and AKD designed the algorithms. FH implemented the algorithms. VU and AKD conceived of the study. FH and AKD drafted the manuscript. BX, WH, JW, and PR helped analyze the results. All authors read and approved the final manuscript.

## Declarations section

Publication of this article was supported by a donation from Molecular Kinetics, Inc.

This article has been published as part of *BMC Bioinformatics *Volume 15 Supplement 17, 2014: Selected articles from the 2014 International Conference on Bioinformatics and Computational Biology. The full contents of the supplement are available online at http://www.biomedcentral.com/bmcbioinformatics/supplements/15/S17.
