# Erratum to: Improving protein order-disorder classification using charge-hydropathy plots

**DOI:** 10.1186/s12859-015-0646-5

**Published:** 2015-07-31

**Authors:** Fei Huang, Christopher J. Oldfield, Bin Xue, Wei-Lun Hsu, Jingwei Meng, Xiaowen Liu, Li Shen, Pedro Romero, Vladimir N. Uversky, A. Keith Dunker

**Affiliations:** Center for Computational Biology and Bioinformatics, Department of Biochemistry and Molecular Biology, Indiana University School of Medicine, Indianapolis, Indiana USA; Department of Cell Biology, Microbiology, and Molecular Biology, University of South Florida, Tampa, Florida USA; Chemical and Biological Engineering, University of Wisconsin-Madison, Madison, Wisconsin USA; Department of Molecular Medicine, University of South Florida, Tampa, Florida USA; USF Health Byrd Alzheimer’s Research Institute, Morsani College of Medicine, University of South Florida, Tampa, Florida USA; Institute for Biological Instrumentation, Russian Academy of Sciences, 142290 Pushchino, Moscow Region, Russia

During the production of our manuscript [1], an incorrect Figure 1 was put in place of the one we submitted, and we erred in not appropriately informing the production staff of this mistake. The corrected figure and the text describing this figure, as well as the figure legend, are present herein.

**Figure 1.**
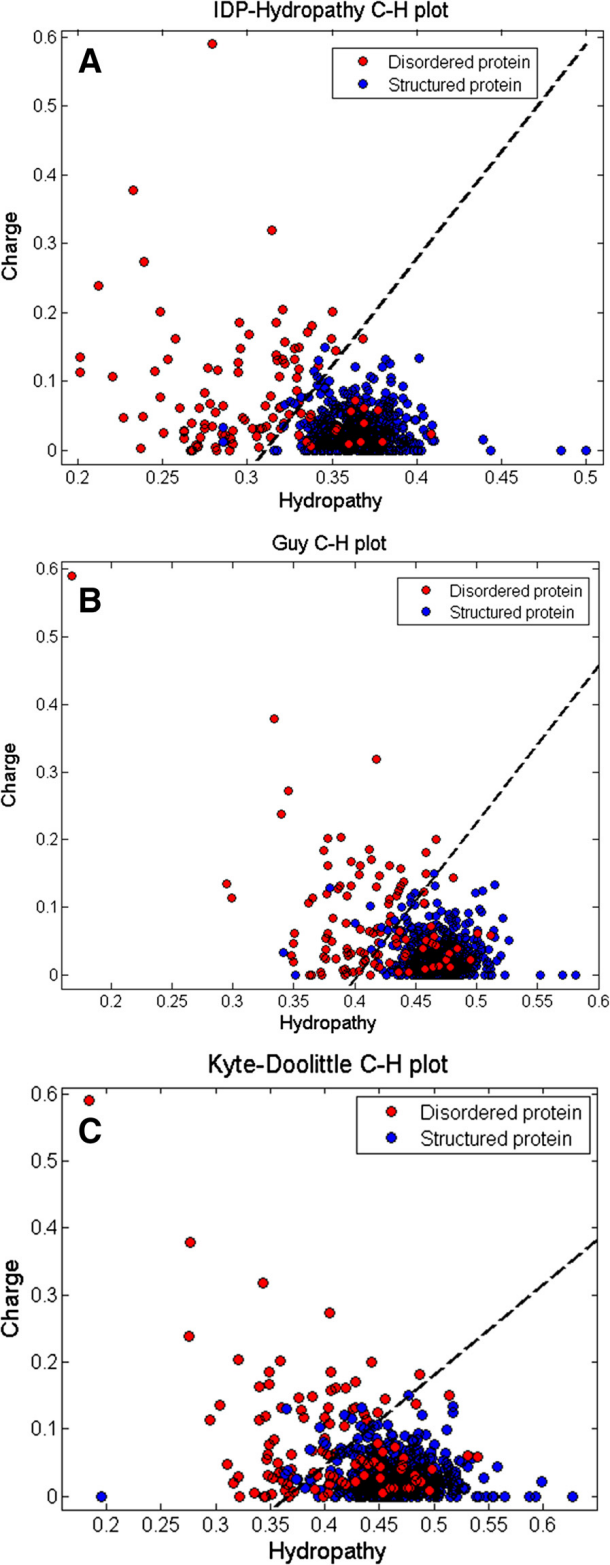
**Charge-Hydropathy plots.** In (**A**) the IDP-Hydropathy scale was used, in (**B**) the Guy (1985) Hydropathy scale was used, and in (**C**) the Kyte-Doolittle (1981) hydropathy scale was used. Red circles indicate disordered proteins, blue circles indicate structured proteins. For these plots, each scale was normalized to be in the interval of 0 to 1. The Guy’s scale is multiplied by -1 prior to normalization to conform to the energy rule set by Kyte-Doolittle scale. In (**A**) the function describing the boundary is: <charge> = 3.31 <hydropathy> 0.97. In (**B**) the function describing the boundary is: <charge> = 2.32 <hydropathy> 0.93. In (**C**), the function describing the boundary is: <charge> = 1.35 <hydropathy> 0.49.

As stated in our manuscript [1], “The C-H plots generated using scale SVM parameters scale, Kyte-Doolittle hydropathy scale, and Guy hydropathy scale for whole protein prediction are shown in Figure 1. Figure 1A, which is derived by SVM parameters scale, shows many fewer misclassified disordered proteins on the ordered side, compared to Figure 1B and 1C.”

As further stated in reference [1]: “Figure 1. Charge-Hydropathy plots. In (A) the IDP-Hydropathy scale was used, in (B) the Guy (1985) Hydropathy scale was used, and in (C) the Kyte-Doolittle (1981) hydropathy scale was used. Red circles indicate disordered proteins, blue circles indicate structured proteins. For these plots, each scale was normalized to be in the interval of 0 to 1. The Guy’s scale is multiplied by -1 prior to normalization to conform to the energy rule set by Kyte-Doolittle scale. In (A) the function describing the boundary is: <charge> = 3.31 < hydropathy> -0.97. In (B) the function describing the boundary is: <charge> = 2.32 < hydropathy> -0.93. In (C), the function describing the boundary is <charge> = 1.35 < hydropathy> -0.49.”

For more details, the reader is referred to the published manuscript [1].
